# Variables Associated With In-Hospital Lethality in COVID-19: A Prospective Cohort Study From Colombia

**DOI:** 10.7759/cureus.69368

**Published:** 2024-09-13

**Authors:** Alvaro J Lora Mantilla, Catalina Cáceres Ramírez, Andrea K Riaño Duarte, Maria C Amaya Muñoz, Maria C Ayala-Gutierrez, Silvia J Villabona, Julian C Cala Duran, Paul Anthony Camacho López, Edgar D Gomez Laitton

**Affiliations:** 1 Research, Development, and Technological Innovation Department, Fundación Oftalmológica de Santander, Floridablanca, COL

**Keywords:** cohort study, colombia, covid-19, lethality, sars cov-2

## Abstract

Introduction: The emergence of COVID-19 represents the most significant health crisis in recent history. Incidence and mortality rates depend on several factors. Many studies have focused on investigating which characteristics could be strongly related to higher mortality and lethality.

Objective: This study aims to analyze the variables associated with in-hospital mortality among patients admitted in a reference northeastern region of a Colombian institution.

Methods: An ambidirectional cohort, single-center study was carried out in a reference hospital in northeastern Colombia. All patients admitted to the Fundación Oftalmológica de Santander (FOSCAL) between March 2020 and September 2021, with COVID-19 real-time polymerase chain reaction (PCR) positive test, were included.

Results: A total of 3,028 patients were included, of whom 2,034 (67.8%) survived and 994 (32.8%) died during their hospital stay; 48.8% (1,479) of the patients were female. The most common comorbidities were hypertension (1,236 patients, 40.8%), obesity (body mass index (BMI) ≥ 30; 656 patients, 21.6%), and diabetes (618 patients, 20.4%). The average age of the surviving patients was 52.2 years, while for the deceased patients, it was 70.3 years. The variables that showed significant association with in-hospital mortality were as follows: male sex ≥ 45 years, dyspnea, oxygen saturation (SatO2) < 85%, hypertension, chronic kidney disease (CKD), and a Charlson Comorbidity Index (CCI) score of >1.

Conclusions: Male sex, age ≥ 45 years, dyspnea, SatO2 < 85%, hypertension, CKD, and a CCI score of >1 were associated with a higher risk of in-hospital mortality in COVID-19-infected patients.

## Introduction

The emergence of COVID-19 represents the most significant health crisis in recent history; SARS-CoV-2 quickly disseminated across the world following the initial reported cases in Wuhan, China, in December 2019. The virus exhibited a distinctive exponential growth pattern, which resulted in the declaration of a global pandemic within a remarkably short period [1]. Now, as of August 8, 2023, the global count of confirmed cases reaches a staggering figure of 768,982,331 million [2,3]. In Colombia, the first confirmed case of COVID-19 was reported on March 6, 2020, followed by the first reported death attributed to COVID-19 infection on March 21, 2020. Currently, in our country, the coronavirus figures reported for June 7, 2023, have confirmed 6,369,916 cases, of which 1,625 are active (have not recovered from the SARS-CoV-2 infection) and 142,780 have been reported as dead from this disease [4].

Prevalence and mortality rates of COVID-19 depend on several factors that include the geographic location, rate of transmission, percentage of population susceptible to infection, effectiveness of community preventive measures, and the strength of healthcare system. Numerous studies have focused on investigating which demographic characteristics, comorbidities, signs and symptoms, laboratory parameters, and image predictors could be strongly related to a higher probability of mortality and lethality [5]. Some studies have addressed the issue of the factors associated with the lower survival of patients with COVID-19 in Colombia. Malagón-Rojas et al. [6] sought to delve into the mortality and survival rates of COVID-19 cases in Colombia between March and July 2020, taking into account the pre-existing chronic clinical conditions that had been related to a greater risk of developing acute syndrome respiratory stress and death. They found that the variables significantly associated with dead are as follows: male sex, >60 years, and hospitalization in Barranquilla, Cartagena, or the Atlántico Department. Another significant contribution came from Rodríguez Lima et al. who focused on describing the clinical characteristics associated with the mortality rates of patients with COVID-19 in a high-complexity hospital in Bogotá. They showed that older age, arterial blood oxygen saturation to low fraction of inspired oxygen (SpO2/FiO2) ratio, and high low-density lipoprotein (LDH) at admission were predictors of mortality during hospitalization [7].

These publications represent important contributions to the detailed analysis of the conditions that may be related to a lower probability of in-hospital survival in patients with COVID-19. Despite this, to date, no article has been published that examines the survival behavior of patients in the northeastern region of Colombia. Therefore, this article represents a significant contribution to the understanding of the behavior of COVID-19 in diverse population groups within our country, considering the different altitudes, as Bucaramanga is located at 959 meters above sea level (masl).

In this way, the aim of this study is to analyze the variables associated with in-hospital mortality among patients admitted in a reference northeastern region of Colombian institutions. This could provide knowledge for planning intervention measures for a resurgence of respiratory infection diseases.

## Materials and methods

Study design and participants

An ambidirectional cohort, single-center study was carried out in a reference hospital in northeastern Colombia. The baseline characteristics of the cohort were previously published [8]. All patients admitted to the Fundación Oftalmológica de Santander (FOSCAL) (Floridablanca, Colombia), between March 29, 2020, and September 27, 2021, with COVID-19 real-time polymerase chain reaction (PCR) positive test, were included. The exclusion criteria were as follows: patients who did not have medical records (those who presented at the emergency room (ER) without vital signs or who died prior to being admitted and assessed by a physician or patients with postmortem diagnosis), patients with incomplete medical records (records in which the absence or presence of any data that was investigated in the survey could not be determined), patients who refused to participate, and alive patients who could not be contacted. The Research Ethics Committee of the FOSCAL Clinic approved this study (02895/2020), and electronic informed consent was obtained from all study participants.

Study setting

FOSCAL is a designated hospital for patients with COVID-19 located in Floridablanca (Metropolitan area of Bucaramanga), Colombia. Bucaramanga is the capital of the northeast province of Santander, surrounded by multiple rural towns and situated 3,146 feet (959 m) above sea level. FOSCAL is a tertiary private hospital facility that includes an emergency department equipped with 14 medical offices and 50 emergency beds. It also houses 499 hospital beds and has the capacity for 210 two-patient rooms for hospitalization. In addition, there are 34 intensive care unit (ICU) beds available at FOSCAL, along with an additional 56 beds at FOSCAL International.

Data collection

Trained research personnel performed collection of the data using the electronic medial records of all patients; for those who were discharged alive, also retrospective telephone interviews were performed to validate and complete the information. LimeSurvey (electronic survey software) was used to manage the data to minimize missing entries and facilitate real-time data validation and quality control [9]. Patients were enrolled into the study since their admission and were followed until either death or discharge from hospital. Demographic information, comorbidities (documented through the International Classification of Diseases, 10th Revision (ICD-10) diagnosis codes), outpatient medication (including self-medicated therapies for the prevention or symptomatic management of COVID-19), smoking, clinical presentation, vital and physical signs at the ER, and discharge outcomes (discharge alive or death) were recorded for all patients.

For hospitalized patients, the following variables were also collected: length of hospital stay, need for ICU management, patient severity assessment, clinical laboratory results, diagnostic imaging results, in-hospital pharmacological treatment, and hospital complications. 

Charlson Comorbidity Index (CCI)

The CCI is a weighted index that uses the number and severity of various comorbidities that may increase mortality risk over the next decade and calculate a score based on the total weights linked with the patient's comorbidities [10]. It has been previously validated in a Colombian population using medical records [11]. The CCI score is classified into three categories: 0-1, low mortality risk in 10 years; 2-5, moderate mortality risk in 10 years; and ≥6, high mortality risk in 10 years. CCI was determined using the Charlson et al. grading system [12].

Procedures and outcome data

The clinical outcomes assessed were the length of stay and discharge or death after hospitalization. 

Statistical methods

We used STATA 16.1 (Released 2020; StataCorp LLC, College Station, Texas, United States) to perform our analysis. Descriptive data for continuous variables are presented as mean with standard deviation (SD) for variables with normal distribution, median with interquartile range for those without normal distribution, and absolute values and percentages for categorical variables. Participants were categorized by treatment location into general hospitalization and ICU care. 

Kaplan-Meier survival analysis was conducted to compare survival rates among different age groups, patients with cancer, hypertension, diabetes, and chronic obstructive pulmonary disease (COPD). Furthermore, the association between hospital stay duration and CCI was examined across various types of hospital stays. Statistical significance was assessed using the log-rank statistic.

A maximum likelihood (ML) binomial regression model with Broyden-Fletcher-Goldfarb-Shanno (BFGS) optimization technique was employed to achieve convergence in the analysis. The results, presented as risk ratios (RRs) with a 95% confidence interval (CI), evaluated the association between demographic and clinical characteristics, and the overall risk of death in hospitalized patients with COVID-19. Initially, an unadjusted analysis was performed without considering confounding factors (sex and age), followed by an additional analysis (Model 1) that controlled for these factors. Both analyses utilized maximum likelihood binomial models with BFGS optimization. Through the binomial ML analysis with BFGS optimization, the association between significantly associated variables of interest and the probability of death in hospitalized patients with COVID-19 was estimated. RRs were used to measure the associations, and a 95% CI to assess the precision of the estimates. Significant statistical differences were considered with a p-value of <0.05.

## Results

A total of 3,028 patients were included, of whom 2,034 (67.2%) survived and 994 (32.8%) died during their hospital stay. The cohort exhibited nearly equal gender distribution, with 48.84% (1479) females and 51.16% (1549) males. A total of 42.57% (1289) of the patients were between 45 and 69 years old, 31.87% (965) 70 years or older, and 25.56% (774) between 18 and 44, with a mean age of 58.21 years (SD: 57.52-58.89). The average age of the surviving patients was 52.27 years (SD: 51.47-53.07), while for the deceased patients, it was 70.35 years (SD: 69.45-71.25). Additionally, 12.18% (345) of individuals were healthcare workers. Regarding medical history, it was found that the most common comorbidities were hypertension (40.82%, 1236), obesity (21.66%, 656), diabetes (20.41%, 618), and dyslipidemia (16.12%, 488). The study captured a range of symptoms at admission, with cough (70.08%, 2122) and dyspnea (65.89%, 1995) being the most prevalent. Vital signs at admission also varied, with 34.44% (1002) having a heart rate of >100 bpm and 48.74% (1415) with a respiratory rate of >20 rpm. The majority of patients had SatO2 levels of >= 85 (77.99%, 2268). The study population had an overall CCI of 2.60 (95% CI: 2.51-2.69), with 41.74% (1264) categorized as Charlson 0-1, 44.02% (1333) as Charlson 2-5, and 14.23% (431) as Charlson >= 6 (Table 1). The median in-hospital survival time was 19 days, and the survival rates for days 10, 20, 30, and 60 were 77%, 47%, 26%, and 14%, respectively.

**Table 1:** Sociodemographic characteristics of the included patients

Variables	Total (3028)
Demographic	
Gender	
Female	1479 (48.84%)
Male	1549 (51.16%)
Age	
Mean age	58.21 (57.52-58.89)
18-44 years	774 (25.56%)
45-69 years	1289 (42.57%)
>=70 years	965 (31.87%)
Healthcare worker	345 (12.18%)
Medical history	
Arterial hypertension	1236 (40.82%)
Obesity	656 (21.66%)
Diabetes	618 (20.41%)
Dyslipidemia	488 (16.12%)
Chronic kidney disease	234 (7.73%)
Cancer	
Hematologic	53 (1.75%)
Solid	140 (4.62%)
Metastatic solid	30 (0.99%)
Chronic obstructive pulmonary disease	168 (5.55%)
Current smoking	38 (9.41%)
Symptoms at admission	
Cough	2122 (70.08%)
Dyspnea	1995 (65.89%)
Myalgia	895 (29.56%)
Headache	879 (29.03%)
Odynophagia	706 (23.32%)
Chills	655 (21.63%)
Diarrhea	576 (19.02%)
Ageusia	565 (18.66%)
Arthralgias	508 (16.78%)
Rhinorrhea	499 (16.48%)
Anosmia	606 (20.01%)
Vital signs at admission	
Mean arterial pressure (MAP) < 60	50 (1.72%)
Heart rate > 100 bpm	1002 (34.44%)
Heart rate >20 bpm	1415 (48.74%)
Oxygen saturation	
SatO2 >= 85	2268 (77.99%)
SatO2 70-84	438 (15.06%)
SatO2 <= 69	202 (6.95%)
Retractions	432 (14.68%)
BMI < 19 or > 35	125 (6.50%)
Charlson Comorbidity Index	2.60 (2.51 - 2.69)
Charlson 0-1	1264 (41.74%)
Charlson 2-5	1333 (44.02%)
Charlson >= 6	431 (14.23%)

The RR for in-hospital mortality among male patients compared to female patients was 1.28 (95% CI: 1.16-1.41; p < 0.001). Patients aged 45 to 69 had a 4.64 times higher risk of dead (RR: 4.64; 95% CI: 3.47-6.21; p < 0.001) compared to patients aged 18 to 44; likewise, patients aged 70 years or older had a 9.16 times higher risk (RR: 9.16; 95% CI: 6.90-12.16; p < 0.001). 

Arterial hypertension (RR: 1.11; 95% CI: 1.00-1.23; p = 0.036), diabetes (RR: 1.12; 95% CI: 1.02-1.23; p = 0.014), chronic kidney disease (CKD) (RR: 1.28; 95% CI: 1.16-1.42; p < 0.001), hematological (RR: 1.47; 95% CI: 1.25-1.17; p < 0.001), and metastatic solid cancer (RR: 1.34; 95% CI: 1.07-1.67; p = 0.010) were identified as risk factors for mortality. In contrast, the presence of dyslipidemia was associated with a 17.5% decrease in the risk of mortality (RR: 0.82; 95% CI: 0.73-0.92; p = 0.001). A current smoking history was also associated with a 1.63 times higher risk of mortality (RR: 1.63; 95% CI: 10.83-2.45; p = 0.019). 

Despite cough being the most frequent symptom, it did not show a significant association with in-hospital mortality (RR: 0.93; 95% CI: 0.85-1.02; p = 0.160). However, the presence of dyspnea acted as a significant risk factor for death, with an RR of 1.6 (95% CI: 1.4-1.83; p < 0.001). Myalgia (RR: 0.72; 95% CI: 0.63-0.81; p < 0.001), headache (RR: 0.51; 95% CI: 0.43-0.605; p < 0.001), sore throat (RR: 0.78; 95% CI: 0.68-0.904; p = 0.001), and chills (RR: 0.73; 95% CI: 0.63-0.84; p < 0.001) were associated with a lower risk of mortality. A heart rate of >100 bpm and a respiratory rate of >20 rpm were associated with a higher risk of mortality, with RRs of 1.11 (95% CI: 1.01-1.22; p = 0.023) and 1.41 (95% CI: 1.27-1.56; p < 0.001), respectively. Additionally, an SatO2) of 70%-84% was associated with an RR of 1.56 (95% CI: 1.40-1.72; p < 0.001), while an SatO2of ≤69% was associated with a higher risk of mortality, with an RR of 1.87 (95% CI: 1.69-2.06; p < 0.001). The presence of retractions and a BMI of <19 or >35 also acted as risk factors, with RRs of 1.45 (95% CI: 1.32-1.58; p < 0.001) and 1.99 (95% CI: 1.22-3.23; p < 0.001), respectively.

Patients with a CCI of 2-5 and six or higher had a 1.8 (RR: 1.85; 95% CI: 1.52-2.25; p < 0.001) and two (RR: 2.36; 95% CI: 1.904-2.92; p<0.001) times higher risk of mortality compared to those with an index of 0-1, respectively (Table 2).

**Table 2:** Bivariate analysis by mortality of patients who were hospitalized with COVID-19, age and sex adjusted *Reference group

Variables	Alive (n = 2034)	Deceased (n = 994)	Risk ratio (95% CI)	p-value
Demographic				
Gender				
Female	1100 (74.37%)	379 (25.63%)	*	
Male	934 (60.30%)	615 (39.70%)	1.283 (1.168-1.41)	<0.001
Age				
18-44 years	727 (93.92%)	47 (1.55%)	*	
45-69 years	909 (70.52%)	380 (29.48%)	4.649 (3.478-6.212)	<0.001
>= 70 years	398 (41.24%)	567 (58.76%)	9.163 (6.905-12.16)	<0.001
Healthcare worker	344 (99.71%)	1 (0.29%)	0.028 (0.004-0.2)	<0.001
Medical history				
Arterial hypertension	644 (52.10%)	592 (47.90%)	1.117 (1.007-1.237)	0.036
Obesity	454 (69.21%)	202 (30.79%)	0.958 (0.853-1.075)	0.466
Diabetes	312 (50.49%)	306 (49.51%)	1.122 (1.024-1.23)	0.014
Dyslipidemia	309 (63.32%)	179 (36.68%)	0.825 (0.732-0.929)	0.001
Chronic kidney disease	84 (35.90%)	150 (64.10%)	1.287 (1.163-1.424)	<0.001
Cancer				
Hematologic	26 (49.06%)	27 (50.94%)	1.47 (1.257-1.718)	<0.001
Solid	66 (47.14%)	74 (52.86%)	1.149 (0.994-1.328)	0.060
Metastatic solid	12 (40%)	18 (60%)	1.342 (1.073-1.679)	0.010
Chronic obstructive pulmonary disease	69 (41.07%)	99 (58.93%)	1.073 (0.941-1.223)	0.292
Current smoking	26 (68.42%)	12 (31.58%)	1.63 (1.083-2.454)	0.019
Symptoms at admission				
Cough	1417 (66.78%)	705 (33.22%)	0.934 (0.85-1.027)	0.160
Dyspnea	1184 (59.35%)	811 (40.65%)	1.607 (1.408-1.834)	<0.001
Myalgia	694 (77.54%)	201 (22.46%)	0.722 (0.638-0.816)	<0.001
Headache	760 (86.46%)	119 (13.54%)	0.51 (0.43-0.605)	<0.001
Odynophagia	565 (80.03%)	141 (19.97%)	0.784 (0.68-0.904)	0.001
Chills	507 (77.40%)	148 (22.60%)	0.73 (0.635-0.841)	<0.001
Diarrhea	423 (73.44%)	153 (26.56%)	0.789 (0.692-0.901)	<0.001
Ageusia	499 (88.32%)	66 (11.68%)	0.45 (0.36-0.564)	<0.001
Arthralgias	402 (79.13%)	106 (20.87%)	0.697 (0.591-0.821)	<0.001
Rhinorrhea	398 (79.76%)	101 (20.24%)	0.821 (0.7-0.963)	0.016
Anosmia	534 (88.12%)	72 (11.88%)	0.472 (0.38-0.586)	<0.001
Vital signs at admission				
Mean arterial pressure (MAP) < 60	24 (48%)	26 (52%)	1.09 (0.862-1.38)	0.471
Heart rate > 100 bpm	668 (66.67%)	334 (33.33%)	1.114 (1.015-1.223)	0.023
Heart rate > 20 bpm	795 (56.18%)	620 (43.82%)	1.413 (1.273-1.569)	<0.001
Oxygen saturation				
SatO2 >= 85	1708 (75.31%)	560 (24.69%)	*	
SatO2 70-84	201 (45.89%)	237 (54.11%)	1.56 (1.407-1.729)	<0.001
SatO2 <= 69	53 (26.24%)	149 (73.76%)	1.872 (1.694-2.069)	<0.001
Retractions	174 (40.28%)	258 (59.72%)	1.451 (1.327-1.586)	<0.001
BMI< 19 or > 35	110 (88%)	15 (12%)	1.992 (1.226-3.238)	<0.001
Charlson Comorbidity Index	1.82 (1.73-1.92)	4.18 (4.02-4.33)	1.073 (1.055-1.091)	<0.001
Charlson 0-1	1120 (88.61%)	144 (11.39%)	*	
Charlson 2-5	766 (57.46%)	567 (42.54%)	1.855 (1.525-2.257)	<0.001
Charlson >= 6	148 (34.34%)	283 (65.66%)	2.361 (1.904-2.928)	<0.001

Variables that showed statistical significant correlation with survival were age, comorbidities (hypertension, diabetes, cancer, COPD, ischemic cardiomyopathy, and heart failure) and CCI and SatO2 at ER admission. Sex, BMI, rheumatic disease, and active smoking didn’t show statistical significant differences in survival analysis.

**Figure 1:**
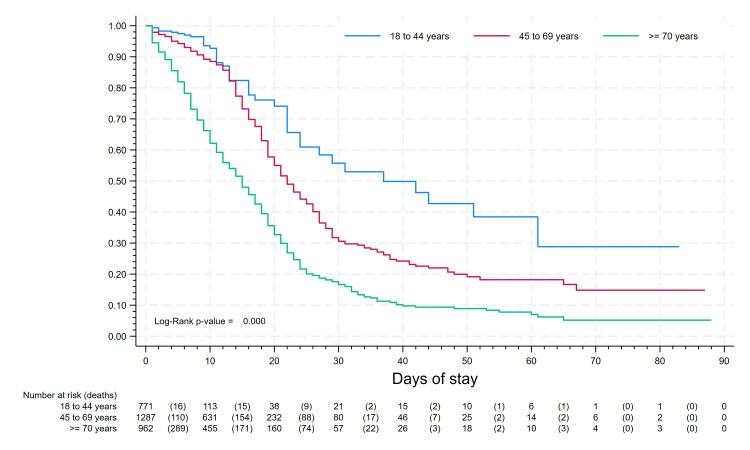
In-hospital surveillance by age group

**Figure 2:**
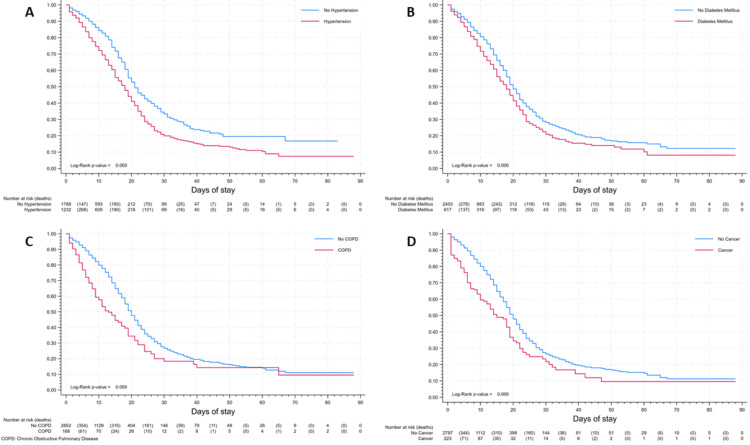
In-hospital surveillance by comorbidities. (A) Hypertension. (B) Diabetes. (C) Chronic obstructive pulmonary disease. (D) Cancer

At day 30, survival rates were 56%, 30%, and 17% for age groups 18 to 44, 45 to 69, and ≥60 years, respectively (Figure 1). In the first 15 days of hospitalization, survival was similar in age groups 18 to 44 and 45 to 69 years; after that, survival rates for these age groups differed until the end of follow-up. In the analysis by comorbidities (Figure 2), at 10 days, survival rates of patients with hypertension, diabetes, cancer, and COPD were 77%, 72%, 60%, and 58%, respectively; comparing with the rates of 85%, 81%, 80%, and 80% in patients without these comorbidities, respectively; additionally, ischemic cardiomyopathy and heart failure also showed statistically significant differences in survival density function analysis.

**Figure 3:**
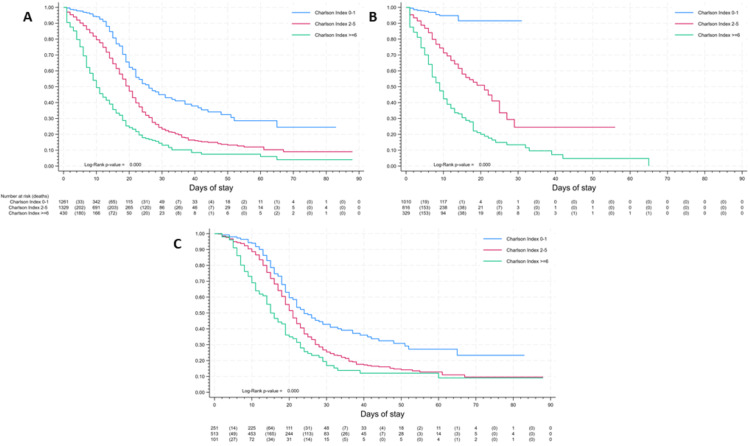
In-hospital surveillance by Charlson Comorbidity Index: (A) General. (B) Patients without ICU admission. (C) Patients with ICU admission ICU: Intensive care unit

CCI scores showed significant correlation with survival that prevails in the categorization by ICU admission (Figure 3). In general, survival rates at 10, 20, and 30 days were 94%, 62%, and 45% for patients with CCI scores of ≤1; 80%, 43%, and 23% for patients with scores between 2 and 5; and 49%, 24%, and 13% for patients with scores of ≥6, respectively (Figure 3A). In patients without ICU admission, at 10, 20, and 30 days, survival rates were 95%, 91%, and 91% for patients with scores of ≤1; 71%, 51%, and 25% for patients with scores between 2 and 5; and 42%, 19%, and 13% for patients with scores of ≥6, respectively (Figure 3B). Finally, in patients with ICU admission, at 10, 20, and 30 days, survival rates were 94%, 60%, and 43% for patients with CCI scores of ≤1; 88%, 52%, and 26% for patients with scores between 2 and 5; and 69%, 35%, and 17% for patients with scores of ≥6, respectively (Figure 3C).

In multivariate binomial regression model analysis, variables that showed significant association with death in COVID-19 hospitalized patients were male sex (RR: 1.772; 95% CI: 1.244, 2.526; p = 0.002), SatO2 between 70 and 84 (RR: 2.71; 95% CI: 1.86-3.95; p = 0.000), SatO2 ≤ 69 (RR: 4.66; 95% CI: 3.15-6.89; p = 0.000), age between 45 and 69 (RR: 2.91; 95% CI: 1.04-8.10; p = 0.041), age ≥ 70 (RR: 4.47; 95% CI: 1.49-13.40; p = 0.007), CCI between 2 and 5 (RR: 2.51; 95% CI: 1.33- 4.74; p = 0.004); CCI ≥ 6 (RR: 4.53; 95% CI: 2.21-9.31; p = 0.000), and BMI < 19 or > 35 (RR: 1.92; 95% CI: 1.20-3.05; p = 0.006) (Table 3).

**Table 3:** Mortality risk ratios in hospitalized Covid-19 patients: a multivariate binomial regression model analysis *Reference group CI: Confidence interval; BMI: body mass index

Variable	RR (95% CI)	p-value
Gender: female	*	
Male	1.772 [1.244, 2.526]	0.002
SatO2 >= 85	*	
SatO2 70-84	2.714 [1.862, 3.956]	<0.0001
SatO2 <= 69	4.663 [3.154, 6.895]	<0.0001
Age: 18-44 years	*	
45-69 years	2.912 [1.046, 8.106]	0.041
>= 70 years	4.479 [1.497, 13.404]	0.007
Charlson Comorbidity Index: 0-1	*	
Charlson 2-5	2.518 [1.336, 4.745]	0.004
Charlson >= 6	4.538 [2.211, 9.317]	<0.0001
BMI: 19-35	*	
BMI < 19 or > 35	1.922 [1.209, 3.057]	0.006

## Discussion

In this cohort study, we examined the clinical characteristics and risk factors associated with in-hospital mortality in patients admitted to the FOSCAL (Floridablanca, Colombia) with COVID-19 real-time PCR positive test; this study included 3,028 patients, 51.16% (1549) were male, and 32.8% (994) died. Male sex, older age, hypertension, diabetes, CKD, cancer, current smoking history, dyspnea, tachycardia, tachypnea, SatO2 < 85%, and a CCI > 1 were significantly associated with increased mortality; additionally, age, hypertension, diabetes, cancer, COPD, ischemic cardiomyopathy, heart failure, and higher CCI were significantly associated with decreased survival rates.

The mean age of surviving patients was 52.27 years (SD: 51.47-53.07), whereas for deceased patients, it was 70.35 years (SD: 69.45-71.25); patients aged 45 to 69 and ≥70 exhibited a 4.6 and nine times higher risk of in-hospital mortality in comparison to patients aged 18 to 44, respectively. These findings were similar to those reported by Malagón et al. who observed that the age group ≥60 years had lower survival rates [6,13]; additionally, a study conducted by Banda et al. at two tertiary-level hospitals in Zambia revealed similar results, demonstrating that older age was associated with nearly double the odds for in-hospital death from COVID-19 [14].

Dyspnea was associated with a higher risk of mortality (RR: 1.607; 95% CI: 1.408-1.834; p < 0.001), affecting 1184 (59.35%) of the survivors and 811 (40.65%) of the deceased. In contrast, anosmia and ageusia were associated with a lower risk of death. Anosmia was present in 534 (88.12%) of the survivors and 72 (11.88%) of the deceased (RR: 0.472; 95% CI: 0.38-0.586; p < 0.001), while ageusia affected 499 (88.32%) of the survivors and 66 (11.68%) of the deceased (RR: 0.45; 95% CI: 0.36-0.564; p < 0.001).

A study conducted by Sarfaraz et al. regarding the determinants of in-hospital mortality in COVID-19 agrees with our findings that dyspnea or shortness of breath (SOB) is the symptom with the highest prevalence in the group of deceased patients, and anosmia had a protective behavior [14,15]; a possible explanation of these findings is that the patients identified anosmia and ageusia as tracer symptoms of COVID-19 infection, and this warrants an early ER consultation. Additionally, a significant proportion of our patients had pre-existing chronic conditions, with the following as the most common: hypertension (1.236 patients, 40.82%), obesity (656, 21.66%), diabetes (618, 20.41%), and a history of smoking (38, 9.41%); current smoking was associated with a 1.63 times higher risk of mortality, findings that are in line with multiple studies, including an Italian cohort study of hospitalized COVID-19 patients, that revealed a significant association between smoking and the risk of mortality [15]. This association is likely due to the induced prothrombotic state that appears to play a crucial role in worse prognosis [16,17].

Our data showed that patients with hypertension (1236 patients, 40.82%) and CKD (234 patients, 7.73%) exhibited a higher relative risk of mortality during their hospital stay; nevertheless, obesity, diabetes, and COPD did not. A previous systematic review conducted by Tamara et al. in 2020 included studies that acknowledged that obesity poses an increased risk of severe COVID-19, being an independent risk factor that significantly impacts the prognosis and necessitates advanced medical treatment for individuals affected by COVID-19. On the other hand, Dessie et al. [12] carried out a meta-analysis showing that mortality among hospitalized COVID-19 patients with diabetes was higher than those patients without diabetes, suggesting that disease is a determinant of severity and mortality of COVID-19 patients, inasmuch as patients with diabetes and COVID-19 often need invasive ventilation care and need ICU due to their likelihood of developing acute respiratory distress syndrome. Despite this, our data contradicts these findings, as we did not find a significant relationship between diabetes (RR: 1.122; 95% CI: 1.024-1.23; p = 0.014) and COPD (RR: 1.073; 95% CI: 0.941-1.223; p = 0.292) with lower in-hospital survival.

Oxygenation indices have been widely studied around the world as determinants of hospital mortality and ICU admission in hospitalized patients with COVID-19; a prospective cohort study from Pakistan showed that SpO2 below 93% confers higher risk of mortality. This parameter serves as an indicator of severe lung involvement and impaired gas exchange. Our multivariate analysis consistently demonstrated that an SatO2 level of <85 was a significant factor associated with lower hospital survival being similar with the mentioned results [14].

The CCI is used to assess the burden of comorbidities. Our analysis showed a significant association with in-hospital mortality. Patients with CCI of 2-5 and ≥6 presented a 1.8 and 1.3 higher risk of mortality compared to those with an index of 0-1, respectively. These results agree with Varol et al. who found that a CCI cutoff value of 2.5 increases the likelihood of mortality 10.7 times. Differences with our findings lie in a smaller sample size (383), lower mortality (8.6%), and therefore a wide CI (OR: 10.5; 95% CI 4.5-25.6) [18]. Despite the above, this confirms that CCI score is an excellent predictor of in-hospital mortality, both inpatients admitted and not to ICU. A study conducted by Gutierrez et al. supports the reliability of the CCI as a tool for predicting the risk of in-hospital mortality, finding that higher scores were associated with lower probabilities of hospital survival [19,20,21].

The multivariate binomial regression analysis revealed a significant association between male sex and mortality in hospitalized COVID-19 patients (RR 1.772; 95% CI: 1.244, 2.526; p = 002). This finding agrees with the results of varied studies [13,15,22,23] which reported differences in both the adaptive and innate immune systems between men and women, specifically within the adaptive immune system, men have lower numbers of CD8+ T cells, CD4+ T cells, and decreased B cell production compared to women. These differences likely contribute to the more favorable prognosis and higher survival rates observed in women with coronavirus infection, as indicated by the analysis of our study [17]. Likewise, two reports from China have highlighted that male individuals with COVID-19 face a higher risk of experiencing unfavorable outcomes and lower chances of survival, irrespective of their age [24-26].

This study had some limitations that should be acknowledged. Firstly, the availability and quality of the data were influenced by the fact that the information was extracted from medical records. Consequently, there is a possibility that certain information may not have been entirely captured in the system or could contain typographical errors that occurred during data entry. Other limitations that may be affecting factors were the exclusion of vaccination and its effects since data collection began early in the pandemic, and in Colombia, the vaccination effort commenced at the end of March 2021, initially targeting medical personnel. Subsequently, vaccination of the general population was implemented in stages based on priority. Therefore, only a minority patients of our cohort were vaccinated, but this study does not reflect the impact of vaccination measures, which could have influenced the outcomes observed. Despite these limitations, the results presented in this study hold value for the analysis of variables associated with COVID-19 survival. This is primarily due to the substantial sample size, which strengthens the study's cohort design. Furthermore, the inclusion of the CCI tool in the analysis enhances the robustness of the results and provides additional support for their validity.

## Conclusions

As a result, male sex, age ≥ 45 years, dyspnea, SatO2 < 85, hypertension, CKD, and a CCI score of >1 were associated with a higher risk of in-hospital mortality in COVID-19-infected patients. We emphasize the value of these findings as potential predictors for our region, and despite the end of the COVID-19 pandemic, these variables can be extrapolated in our population as predictors of mortality in upper respiratory infections that require hospitalization. Therefore, it is necessary to carry out future studies that evaluate its performance in these diseases.
